# Burden of liver cancer resulting from different etiologies in China from 1990 to 2021 and prediction to 2031

**DOI:** 10.1371/journal.pone.0338908

**Published:** 2025-12-18

**Authors:** Meiling Zhang, Yan Tan, Zegui Fang, Feng Li, Xiaoxiao Chen, Xiaoshan Chen, Yongyue Deng, Siyang Huang

**Affiliations:** 1 Department of Infectious Diseases, Zhongshan Hospital Xiamen University, School of Medicine, Xiamen University, Xiamen, Fujian, China; 2 Department of Nephrology, The First Affiliated Hospital of Xiamen University, School of Medicine, Xiamen University, Xiamen, Fujian, China; Baylor Scott & White Research Institute, UNITED STATES OF AMERICA

## Abstract

Liver cancer (LC) imposes a considerable disease burden in China, with diverse causes contributing to varying proportions of LC cases. Adjusting future public health strategies for the allocation of resources to interventions aimed at different causes of LC is a subject that demands resolution in order to maximize health benefits. Employing the Global Burden of Disease (GBD) 2021, we analyzed the burden of LC in China attributed to different causes. Utilize joinpoint to evaluate the trend of the disease burden of LC. The Bayesian model for age-period-cohort (BAPC) was adopted to forecast the burden. The overall trend of the age-standardized rates of incidence, mortality, and disability-standardized life years (ASIR, ASMR, and ASDR) of LC were descend in China from 1990 to 2021. HBV, HCV, alcohol and non-alcoholic steatohepatitis (NASH) constitute the four principal causes of the LC burden. The ASIR, ASMR, and ASDR of LC caused by HBV and HCV showed a downward trend. The ASIR of LC caused by alcohol and NASH were associated with an increased. It was anticipated that the burden of ASIR and ASMR for male, female, and the entire population in China will persistently decline until 2031. In conclusion, the burden of LC has displayed a downward trend in China from 1990 to 2021; HBV, HCV, alcohol, and NASH were the main causes of LC; the burden of LC is expected to further decline by 2031.

## Introduction

In 2022, approximately 865 thousand new cases of liver cancer (LC) emerged worldwide, ranking sixth in terms of cancer incidence; and approximately 7,579,480 deaths occurred, ranking third in terms of cancer mortality [1]. LC was ranked among the top five most prevalent cancers in China and constitutes one of the principal causes of cancer-related deaths [1,2]. From 2000 to 2018, age-standardized rates (ASR) of incidence (ASIR), mortality (ASMR) of LC in China manifested a pronounced downward trend [2]. Nevertheless, considering the vast population base in China, the disease burden remains profoundly severe. A comprehensive grasp of the burden of LC and the temporal variation patterns of its etiology in China will facilitate the formulation of customized and adjustable intervention strategies.

Previous studies primarily centered on the trends of burden of LC ascribed to a single etiology [3,4]. The lack of a comprehensive analysis of all the causes of LC could potentially give rise to the omission of crucial information concerning the burden induced by various causes. The transition from viral to non-viral liver diseases as the principal risk factor for LC exerts an influence on the formulation of screening, prevention, and treatment strategies [5,6]. At present, non-virus-related LC accounts for 20% ~ 30% of the total incidence of LC [7]. With the acquisition of persistent viral response (SVR) and the change of lifestyle and diet habits, it is expected that by 2030, the incidence of non-alcoholic steatohepatitis (NASH) – LC in China will increase by 82% compared with 2016, and the fatality rate will more than double, while NASH will become the primary cause of LC [8]. In May 2024, the Global Burden of Disease (GBD) 2021 updated its data, and the previously studied GBD 2019 data on LC in China has changed [3,9,10]. GBD 2019 classifies the etiologies of LC into five categories [Hepatitis B (HBV), Hepatitis C (HCV), alcohol-related, non-alcoholic steatohepatitis (NASH) and other causes)] [9], whereas GBD2021 has incorporated an additional classification for LC. The other causes of LC include aflatoxin exposure, aristolochic acid, chronic biliary disease, hereditary or metabolic liver disease, etc. At present, a scarcity of comprehensive analyses exists regarding the burden of LC and its new etiological classification in China. Consequently, we opined that it was indispensable to update the data on the burden of LC in China expeditiously, facilitating policymakers to grasp the situation and formulate efficacious prevention and control strategies.

This study aims to comprehensively evaluate the changing trends, etiological composition, and future projections of LC burden in China from 1990 to 2021, providing scientific evidence for optimizing healthcare resource allocation and reducing disease burden. Specific objectives include: (1) Quantifying the incidence, mortality, and disability-adjusted life years (DALYs) burden levels of liver cancer in China during 1990–2021, along with their evolving trends, while analyzing proportional changes caused by different etiologies; (2) Identifying critical turning points and phased trends in the burden of LC and its primary causes (ASIR, ASMR, ASDR) across China from 1990 to 2021; (3) Predicting the trajectory of LC of ASIR, ASMR in China from 2022 to 2031. The core objective of this study is to map the long-term dynamic changes in China’s LC burden, clarify the evolving contributions of major etiologies, and forecast future trends, thereby providing critical data support for developing targeted prevention strategies and optimizing healthcare resource allocation.

## Methods

### Data source

GBD 2021 (https://ghdx.healthdata.org/gbd-2021) is the data source of this study. GBD 2021 was updated on May 16, 2024, and encompasses data regarding 371 disease burdens and diverse risk factors for 204 countries/regions spanning from 1990 to 2021 [11]. The Chinese data of GBD were obtained from the National Disease Surveillance Point System (NDPS), the China Death Cause Monitoring System, the National Cancer Registry (NCR), and the China CDC Death Cause Registration Report Information System. We acquired the data regarding the LC-related burden, including the numbers, rates (per 100,000 population), and ASIR, ASMR, and ASR of DALYs (ASDR) by age and sex in China from 1990 to 2021. Previous articles have offered a meticulous description of the methods employed by the GBD 2021 [12,13]. The prime focus rests upon the employment of comprehensive Meta-regression analysis and Bayesian regression tools for the estimation of the burden. In the GBD LC category, the second-level causes that give rise to LC are categorized into six groups, namely HBV, HCV, alcohol, NASH, hepatoblastoma, and other causes.

We retrieved the GBD 2021 World Population Standard Table and the 2017–2100 World Population Projection Table for the purpose of calculating the number of ASR for each age group, and subsequently employed BAPC to prognosticate the status of LC in China ten years hence.

### Analysis method

Estimated annual percentage change (EAPC) constitutes a highly efficacious and widely employed approach for evaluating trends in incidence, mortality, and other indicators within a specific time frame [14]. Compute the EAPC [95% confidence intervals (CI)] of ASIR, ASMR, and ASDR to quantify the temporal trend of the burden of LC in China from 1990 to 2021. The trends were considered a decrease when the upper boundary of the 95% CI of EAPC was less than 0; whereas if the lower boundary was greater than 0, the upward trends of the burden were defined; otherwise, the trends were stable [14,15].

Joinpoint regression analysis (https://surveillance.cancer.gov/joinpoint) is capable of suggesting a logarithmic regression model based on data characteristics and can analyze the trend features of the rate and normalized rate that vary over time. The specific calculation method has been elaborated in detail [16]. The model can calculate annual percentage change (APC), average annual percent change (AAPC), and their 95% CI. APC indicates the description of the alteration in data within a particular period; AAPC represents the average annual rate of change of data throughout the entire time span to reflect the overall trend of data variance over an extended period.

The BAPC possesses excellent predictive capability, and its predictive accuracy is relatively high within the 15-year forecasting period [17]. To ensure a high level of predictability, accordingly, this article predicted the burden of LC in China ten years. Employing the burden of LC in China from 1990 to 2021 as the test set, the R software BAPC was utilized to predict the variations in ASIR and ASMR for different genders and the entire population during 2022–2031 [9,17].

### Statistical analysis

All statistical analyses and the visualization of results were accomplished through the R software (Version 4.2.0). By employing Joinpoint regression software version 5.2.0, the trend of burden of LC was analyzed. A two-tailed P value < 0.05 was deemed statistically significant.

### Ethical issues

This study excluded patients and the general public, and thus the publication of the results does not contain any information related to patients or the public.

## Results

### The burden of LC attributed to diverse etiologies in China from 1990 to 2021

The numbers and ASR of incidence, mortality, and DALYs of LC and its six causes in China in 1990 and 2021, along with the EAPC from 1990 to 2021, was presented in Table 1. The overall tendency of ASIR, ASMR, and DALYs for LC was downward in China. The ASIR of LC attributed to HBV, HCV, other causes, and hepatoblastoma were declining. The ASIR of LC attributed to alcohol and NASH were showing an upward trend. The ASMR resulting from HBV, HCV, other causes, and hepatoblastoma has presented a downward trend. The ASMR of LC due to NASH increased in China. The ASDR of LC due to alcohol remained stable. The ASIR of LC due to HBV, HCV, alcohol, other causes and hepatoblastoma were decreasing.The ASDR of LC due to NASH remained constant.

**Table 1:** The cases number and ASR of incidence, mortality, and DALYs of LC and its six causes, with EPAC from 1990 to 2021.

Causes		1990		2021		EAPC (95%CI) 1990-2021
		Number (95%UIs)	ASR (95%UIs) per/100,000	Number (95%UIs)	ASR (95%UIs) per/100,000	
Liver cancer	Incidence	96434 (80971 - 113769)	10.58 (8.94 - 12.43)	196637 (158273 - 243558)	9.52 (7.72 - 11.78)	−0.2752% (−0.4152 - −0.135)
	Death	94937 (79884 - 111527)	10.75 (9.12 - 12.61)	172068 (139621 - 212496)	8.35 (6.8 - 10.29)	−0.7633% (−0.9125 - −0.614)
	DALYs	3294864 (2763029 - 3879589)	334.52 (281.08 - 393.14)	4890023 (3905089 - 6124599)	239.91 (191.98 - 299.37)	−1.1605% (−1.3359 - −0.9848)
Liver cancer due to hepatitis B	Incidence	63118 (52018 - 75227)	6.58 (5.45 - 7.84)	118665 (92280 - 153556)	5.73 (4.48 - 7.38)	−0.4199% (−0.5774 - −0.2623)
	Death	61415 (50743 - 73122)	6.53 (5.42 - 7.76)	100194 (77721 - 129138)	4.83 (3.76 - 6.19)	−0.9722% (−1.152 - −0.7921)
	DALYs	2236077 (1842616 - 2663358)	220.05 (181.34 - 260.91)	3148553 (2442865 - 4109014)	155.81 (121.32 - 201.99)	−1.223% (−1.412 - −1.0338)
Liver cancer due to hepatitis C	Incidence	14422 (11688 - 17539)	1.94 (1.57 - 2.32)	36427 (28404 - 44840)	1.78 (1.41 - 2.18)	−0.1487% (−0.2642 - −0.0331)
	Death	15268 (12408 - 18500)	2.16 (1.77 - 2.6)	34899 (27413 - 42965)	1.74 (1.38 - 2.12)	−0.5767% (−0.7067 - −0.4466)
	DALYs	386481 (309229 - 471854)	46.48 (37.56 - 56.75)	751020 (585482 - 933695)	35.49 (27.67 - 44.05)	−0.8295% (−0.9521 - −0.7067)
Liver cancer due to alcohol use	Incidence	7500 (5772 - 9563)	0.84 (0.66 - 1.07)	20464 (15239 - 27296)	0.94 (0.71 - 1.25)	0.6175% (0.4253 - 0.81)
	Death	7575 (5858 - 9677)	0.87 (0.69 - 1.1)	18317 (13653 - 24252)	0.85 (0.64 - 1.12)	0.1591% (−0.0028 - 0.3212)
	DALYs	227509 (174534 - 293034)	24.26 (18.72 - 31.11)	477847 (352518 - 637755)	22.01 (16.3 - 29.15)	−0.176% (−0.3386 - −0.0131)
Liver cancer due to NASH	Incidence	4057 (3237 - 4978)	0.48 (0.38 - 0.58)	11293 (8663 - 14314)	0.54 (0.42 - 0.68)	0.7225% (0.5313 - 0.914)
	Death	4128 (3293 - 5068)	0.5 (0.4 - 0.61)	10409 (8036 - 13180)	0.51 (0.39 - 0.64)	0.332% (0.1415 - 0.5228)
	DALYs	125153 (100593 - 153269)	13.39 (10.77 - 16.4)	256209 (194368 - 326023)	12.22 (9.41 - 15.44)	−0.1574% (−0.3538 - 0.0394)
Liver cancer due to other causes	Incidence	5068 (4017 - 6305)	0.54 (0.43 - 0.66)	9235 (7034 - 11875)	0.45 (0.34 - 0.57)	−0.4613% (−0.5963 - −0.326)
	Death	4981 (3957 - 6185)	0.54 (0.43 - 0.67)	8033 (6102 - 10231)	0.39 (0.3 - 0.49)	−0.924% (−1.0662 - −0.7815)
	DALYs	180928 (145486 - 225056)	17.74 (14.16 - 21.98)	237416 (180748 - 310459)	11.82 (9.11 - 15.35)	−1.3667% (−1.5402 - −1.1929)
Hepatoblastoma	Incidence	2269 (1881 - 2784)	0.21 (0.17 - 0.25)	553 (391 - 783)	0.08 (0.06 - 0.11)	−3.9014% (−4.3629 - −3.4376)
	Death	1570 (1305 - 1924)	0.14 (0.12 - 0.17)	216 (155 - 302)	0.03 (0.02 - 0.04)	−5.9432% (−6.4806 - −5.4026)
	DALYs	138716 (115370 - 169802)	12.6 (10.48 - 15.42)	18978 (13614 - 26641)	2.56 (1.83 - 3.63)	−5.9314% (−6.4662 - −5.3936)

Abbreviations: ASR: age-standardized rates; UIs: uncertainty intervals; EAPC: Estimated annual percentage change; CI: confidence interval; DALYs: disability-standardized life years; NASH: non-alcoholic steatohepatitis.

### The composition of ASIR, ASMR, and ASDR of LC due to various causes

**Fig 1:**
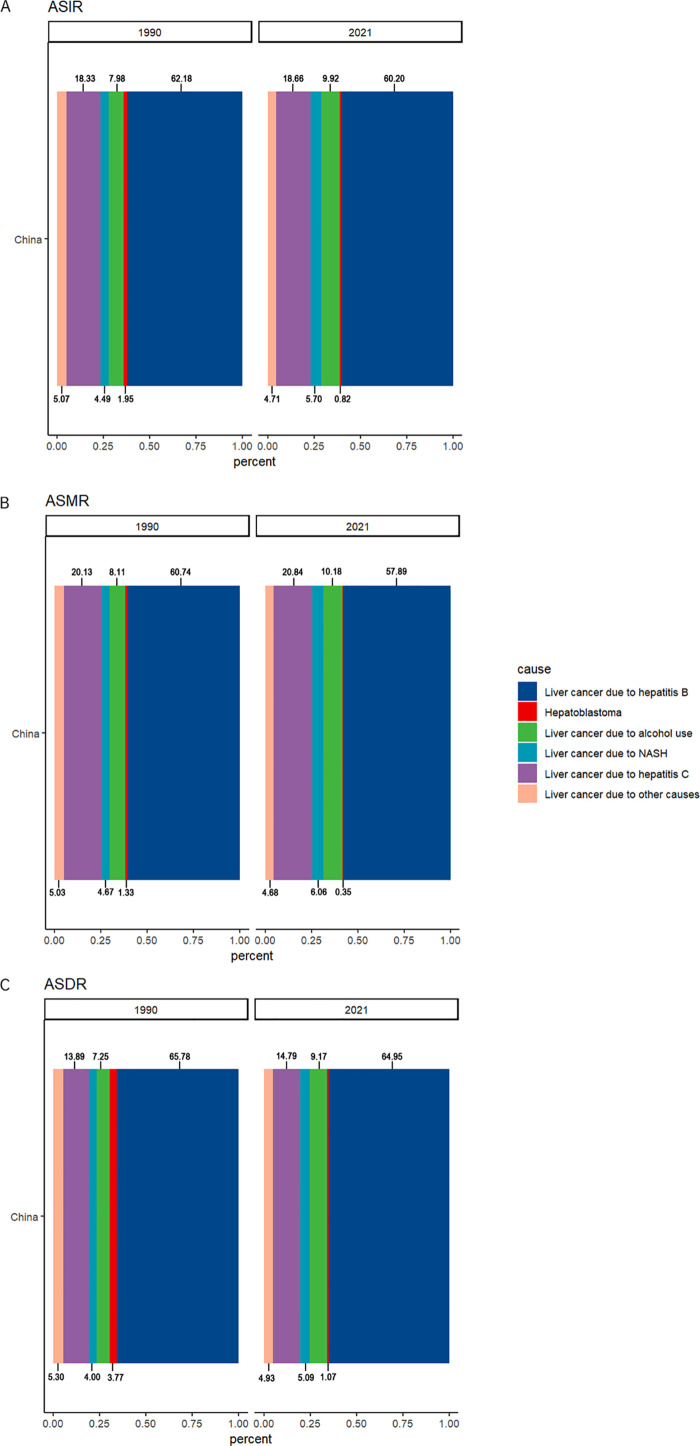
The constitution of the burden of LC caused by various causes in China. **(A) The constitution of ASIR of LC caused by various causes in China. (B) The constitution of ASMR of LC caused by various causes in China. (C) The constitution of ASDR of LC caused by various causes in China.** Abbreviations: ASIR: age-standardized rates of incidence, ASMR: age-standardized rates of mortality; ASDR: age-standardized rates of DALYs; NASH: non-alcoholic steatohepatitis.

HBV, HCV, and alcohol had consistently constituted the three most significant causes of LC in terms of burden in China (Fig 1). In 1990, ASIR accounting for 62.18%, 18.33%, and 7.98%, respectively. In 2021, ASIR ratios were 60.2%, 18.66%, and 9.92%. In 1990, ASMR accounting for 60.74%, 20.13%, 8.11%. In 2021, ASMR accounting for 57.89%, 20.84%, 10.18%, respectively. In 1990, ASDR ratios were 65.78%, 13.89%, 7.25%. In 2021, ASDR accounting for 64.95%, 14.79%, 9.17%, separately.

### Trends of the burden of LC using joinpoint regression analysis

Between 1990–2021, the ASIR, ASMR, ASDR of LC decreased in China (Table 2). The ASIR, ASMR, and ASDR slightly decreased in LC due to HBV, HCV. (P value <0.05). Nevertheless, the ASIR for LC resulting from alcohol and NASH ascended (P value <0.05).

**Table 2:** The joinpoint regression analysis on ASIR, ASMR, ASDR of LC and causes.

cause	ASIR_AAPC	P-Value	ASMR_AAPC	P-Value	ASDR_AAPC	P-Value
Liver cancer	−0.312 (−0.39 - −0.234)	< 0.001	−0.677 (−1.252 - −0.099)	0.022	−0.959 (−1.358 - −0.558)	< 0.001
Liver cancer due to alcohol use	0.384 (0.22 - 0.549)	< 0.001	0.01 (−0.439 - 0.46)	0.967	−0.247 (−0.615 - 0.122)	0.189
Liver cancer due to hepatitis B	−0.42 (−0.512 - −0.327)	< 0.001	−0.925 (−1.362 - −0.486)	< 0.001	−0.989 (−1.461 - −0.515)	< 0.001
Liver cancer due to hepatitis C	−0.248 (−0.388 - −0.108)	0.001	−0.581 (−1.029 - −0.13)	0.012	−0.786 (−1.294 - −0.275)	< 0.003
Liver cancer due to NASH	0.441 (0.343 - 0.539)	< 0.001	0.127 (−0.356 - 0.612)	0.607	−0.23 (−0.71 - 0.252)	0.349
Liver cancer due to other causes	−0.559 (−0.662 - −0.457)	< 0.001	−1.046 (−1.564 - −0.525)	< 0.001	−1.151 (−1.689 - −0.611)	< 0.001
Hepatoblastoma	−2.941 (−3.426 - −2.452)	<0.001	−4.833 (−5.26 - −4.404)	< 0.001	−4.832 (−5.245 - −4.417)	< 0.001

Abbreviations: APC: annual percentage change; AAPC: average annual percent change; NASH: non-alcoholic steatohepatitis.

### Prediction of LC-related burden in china in the next 10 year by sex

**Fig 2:**
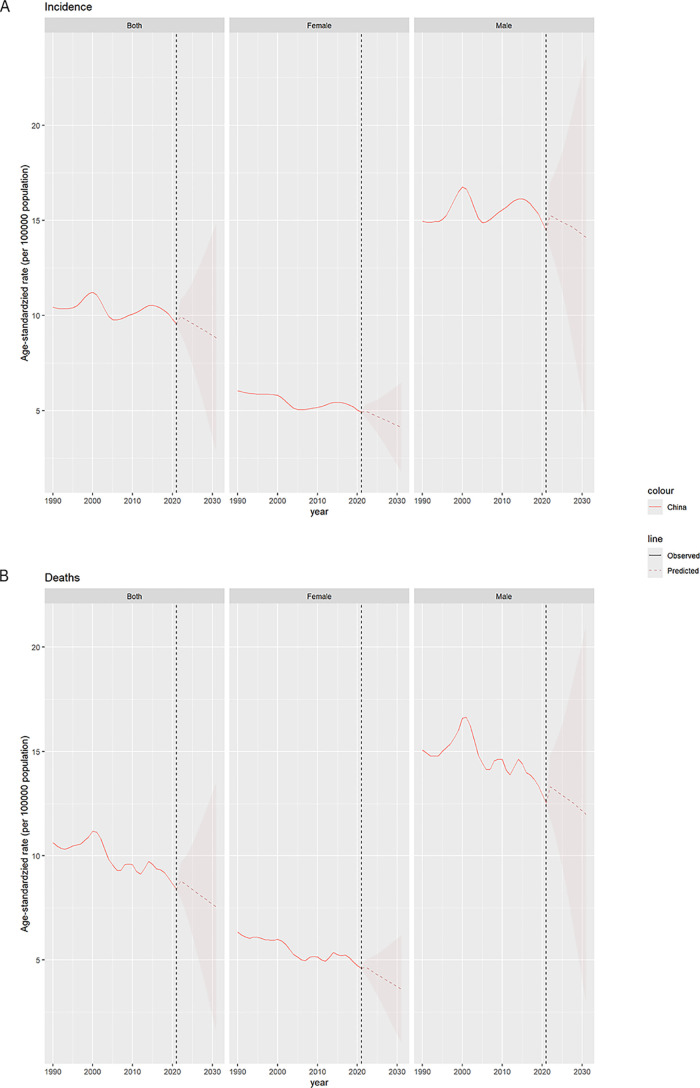
(A) The predicted ASIR of LC to 2031. **(B) The predicted ASMR of LC to 2031.** Abbreviations: ASIR: age-standardized rates of incidence, ASMR: age-standardized rates of mortality.

By employing BAPC, the ASIR and ASMR of LC in China were predicted until 2031 by sex, and the results were presented in Fig 2.

By 2031, the ASIR of LC for male [14.10, (4.44, 23.76)], female [4.13, (1.77, 6.48)], and the overall population [8.82, (2.74, 14.91)] will decrease (S1 Table). Meanwhile, the ASMR (95%CI) of LC for male [11.98, (2.86, 21.09)], female [3.61, (1.01, 6.20)], and the overall population [7.53, (1.46, 13.60)] will decrease either (S2 Table).

## Discussion

Based on the GBD 2021, this study disclosed the burden of LC and its diverse causes in China over the past three decades, and meanwhile provided projections until the year 2031. From 1990 to 2021, the burden of LC decreased in China, which was consistent with the changes on a global scale [1]. The three primary causes of the burden of LC in China persist as HBV, HCV, and alcohol. The burden of LC attributed to HBV and HCV had been consistently decreasing, yet ASIR of LC due to alcohol and NASH had been steadily ascending. From 2022 to 2031, it was projected that the ASIR and ASMR of LC in China will decline steadily.

LC was among the most prevalent types of cancers in China and constituted one of the principal causes of cancer-related fatalities among Chinese male [1,3]. In 2021, HBV was still the leading cause of LC in China, with ASIR, ASMR, and ASDR of LC due to HBV collectively making up about two-thirds of all cases. This finding underscores the continued central role of HBV prevention and control in China’s LC prevention strategy. Although we observe the growing importance of metabolic risk factors, controlling the burden of HBV related LC remains a top priority in the short term. According to statistical data, the number of deaths associated with HBV witnessed an increase of 5.9% from 1990 to 2019, and a rise of 2.9% from 2015 to 2019 in global [18]. China is globally the nation with the severest HBV infection and a major contributor to achieving the goal of eliminating HBV worldwide by 2030 [19,20]. With a deepening understanding of chronic hepatitis B, the number of treated individuals is rising, and improved access to HBV antiviral drugs, along with early LC screening, detection, and treatment, is crucial for reducing the burden [21]. Through concerted efforts, both the burden and the proportion of LC due to HBV has manifested a downward tendency. On the one hand, due to the large population base of chronic HBV infections in China in the past, this group is entering the high-risk age for LC. On the other hand, due to varying economic and cultural development across regions, the diagnosis and long-term standardized treatment of HBV still face severe challenges in adjustment. To achieve the goal of global elimination of HBV by 2030, China has promoted the timely vaccination of newborns to prevent mother-to-child transmission and has ensured widespread coverage of childhood vaccination, with coverage rates exceeding 95% [19,20]. This had resulted in a significant downward trend in the incidence of HBV infection in China. China has accomplished the target set by WHO GHSS for children under the age of 5, with an HBsAg positivity rate of less than 1% by 2020 [18]. Experts anticipate that the number of patients infected with HBV in China will significantly decrease, and the burden of LC caused by HBV will continue to decline. In 2021, China estimated 43.3 million HBV infections, comprising 3% of its total population [20]. Over an extended period in the future, HBV will continue to be the predominant cause of LC in China. The current policy focus needs to shift from preventing new infections to strengthening screening, diagnosis and lifelong treatment of chronic infections in middle-aged and elderly people.

The second major cause of the burden of LC in China was HCV. HCV is predominantly transmitted through blood transmission, sexual transmission and vertical transmission [22]. China has managed to control the spread of HCV through conducting pre-transfusion screening and discontinuing paid blood donation [23]. However, the rampant proliferation of drugs and the growing incidence of male-to-male sexual behavior have presented challenges to the control of HCV. Significant progress has been made in direct-acting antivirals (DAAs) therapy for HCV. The enhanced accessibility of DAAs, shorter treatment periods, the DAAs of sustained virological response >90%, and the simplification of dosing regimens have facilitated the effective treatment of patients with HCV [24,25]. Excluding developed countries, the incidence of LC caused by HCV was gradually diminishing [26]. This accorded with the trend of the decreasing ASIR, ASMR, and ASDR for LC attributed to HCV in China. The intricate biology and the capacity of HCV to evade the immune system have presented challenges to the development of vaccine [27,28], making primary prevention of HCV very difficult. HCV infection typically exhibits no clinical symptoms prior to the manifestation of severe liver disease, thereby leading to the undetected condition of a considerable number of HCV individuals. In 2020, the global prevalence of HCV was 0.7% (56.8 million cases), with an estimated one quarter of the infected individuals have been diagnosed and only 641,000 have commenced treatment [29]. The expense of DAAs is considerable, which poses a challenge for a substantial number of individuals to access HCV treatment. Furthermore, the refractory of HCV persistently impose challenges upon the DAAs [24]. As compared with 1990, HCV contributed to an increase in the ASIR, ASMR, and ASDR of LC in China. In 2019, China’s DAAs were officially included in the medical insurance. After medical insurance reimbursement, the cost of drugs for patients was greatly reduced, which improved the cure rate of HCV patients. Researchers have steadily made progress in the development of innovative vaccine candidates, offering hope for alleviating the burden of HCV [27,28]. In the future, efforts need to be intensified for the further development of HCV vaccines to reduce the infection rate and speed up the spread of DAAs, allowing HCV to receive treatment promptly.

Emerging risk factors, such as metabolic disorders (for instance, alcoholic liver disease and NASH), are impeding the downward trend of LC incidence and mortality rates [1,3,5]. Alcohol has caused an increase in the ASIR of LC in China, and the burden of LC due to alcohol has increased proportionately, from 1990 to 2021. Over the past two decades, the burden of alcohol-related liver disease among adolescents and young adults aged 15–29 worldwide has witnessed a notable increase [30], the situation of alcohol abuse demands serious attention. NASH played a contributory role in the increasing incidence rate of LC in China, and the proportion of the burden was steadily increasing. During the past four decades, the number of overweight and obese individuals in China has undergone a dramatic increase [31]. NASH is a disorder that is closely associated with obesity and metabolic syndrome. The LC caused by NASH has seen the most prominent increase in ASMR across regions worldwide [26]. Currently, no specific drug therapy for NASH has been approved. The main treatment for NASH is lifestyle changes through diet and exercise, including low calorie diet, moderate exercise, treatment of metabolic complications, and smoking and alcohol abstinence [32]. It is essential to establish public health policies aimed at reducing alcohol abuse and facilitating weight control in order to prevent the emergence of alcohol-related liver disease and NASH. The current screening indicators demonstrate low sensitivity in the detection of early LC among patients with non-viral liver diseases [5], and highly sensitive and specific screening indicators need to be identified. At present, GALAD score shows strong advantages in the early diagnosis of Hepatocellular Carcinoma (HCC) in patients with NASH and can monitor the occurrence of HCC in early [33]. Risk prediction models for NASH related LC in cirrhotic patients include the ADRESS-HCC risk model, Toronto HCC Risk Index, and APAC risk index. For non-cirrhotic patients, established models such as the polygenic risk score (PRS) and PRS-HFC score have been developed. However, these predictive models require extensive validation through prospective cohort studies across diverse populations to confirm their clinical applicability [34].

We projected that ASIR and ASMR of LC in China will continue to decline by 2031. Notably, the ASIR and ASMR of male patients with LC in China were strikingly higher than female and the entire population. This conformed to the upward trend of ASIR of LC among male aged 60 and above on a global scale [26]. Estrogen can exert a protective impact on LC development via estrogen receptor alpha-mediated signal transduction, and androgen can contribute to LC development, thereby giving rise to a higher incidence of LC in male when compared with female [35]. For male prone to the risk of LC, the early implementation of intervention measures, the enhanced screening efforts, or the elevated screening frequency can yield more substantial benefits in reducing the risk of LC or facilitating early detection. LC is not merely correlated with viral infections, but intricately associated with a multiplicity of risk factors, such as obesity, unhealthful dietary habits, alcohol consumption, and metabolic disorders [5]. Due to the evolving etiologies of LC, the target population for the screening of LC is becoming increasingly challenging to capture [6]. Presently, ultrasound (US) and AFP constitute the predominant screening modalities for LC, and early diagnosis of LC is capable of enhancing survival rates [21,36]. At present, no unambiguous recommendation concerning screening frequency is available. Studies have demonstrated that screening every 3–4 months can identify a greater number of early-stage LC cases; nevertheless, there was no discernible increase in survival benefit when contrasted with screening cycles of 6–12 months [21]. When Computed Tomography (CT) and Magnetic Resonance Imaging (MRI) are utilized as screening modalities, they are concomitant with radiation exposure and considerable costs. Nonetheless, for patients afflicted with cirrhosis, surveillance through MRI is capable of detecting LC at an earlier stage compared to US, presenting a higher detection rate and a lower false positive rate, which is expected to augment the clinical outcomes of LC [37]. The screening for LC within high-risk is evidently more beneficial rather than risky [21,38]. By establishing risk prediction models to identify the groups for LC screening could be defined more rationally that can get the greatest benefits [6]. The study suggested a risk assessment algorithm using multiple biomarkers and clinical indicators, along with a stepwise screening strategy, which is now seen as a good way for early-stage LC screening [6]. The selection of appropriate screening target has the capacity to diminish unnecessary screening expenses and averting unnecessary radiation exposure, thereby attaining the utmost benefit in the allocation of health resources.

The following limitations exist in this study: (1) The restricted information regarding LC from the GBD 2021 constrain the burden analysis of LC in this study, despite GBD 2021 has refined the model to enhance the accuracy of the estimates; (2) Owing to the information latency (currently updated merely to 2021), the trends of burden of LC in China were evaluated based on the current data, and the predicted results might potentially be inaccurate; (3) The GBD 2021 is deficient in LC data for each region in China, thereby rendering it infeasible to undertake further analysis of the geographical variances in LC.

Despite the decrease in the ASIR, ASMR, and ASDR of LC, due to the large population base, LC remains a major public health concern in China. Through efforts made in recent years, the burden of LC due to HBV, HCV has been effectively managed and controlled. Nevertheless, HBV and HCV constitute the most prominent causes of LC. The proportion of LC due to alcohol and NASH was steadily rising. This suggested that as the etiological composition of LC changes, it emphasized the need for new requirements to control and manage the risk factors. These findings may provide helpful guidance for making better public health policies and allocating medical resources. Health policy makers in China are obliged to further deliberate on targeted measures and flexible approaches with the aim of enhancing the personalized medical care system.

## Supporting information

S1 Table Prediction of ASIR of LC in China. (XLSX)

S2 Table Prediction of ASMR of LC in China. (XLSX)

S1 File LC_china_sex. (XLSX)

S2 File Liver_cancer. (XLSX)
